# A Case of Ascites of Long Standing

**Published:** 1856-06

**Authors:** E. W. Cheney

**Affiliations:** Canandaigua, N. Y.


ART. III.—*A Case of Ascites of long standing.* By E. W. CHENEY, M. D., Canandaigua, N. Y.

The following case has no particular interest except from the amount of fluid evacuated, and the facility with which nature accommodates herself to varying circumstances:

On the 4th day of October, 1855, I was called to visit Mrs. P., a widow lady, about 45 years of age, in the town of Hopewell, Ontario county, who was laboring under ascites of long standing.

I found the abdomen immensely distended, so much so that fatal interference with vital organs seemed imminent. The lower extremities were considerably distended, but the upper were wholly free from bloating. The abdomen had been enlarging for the last ten or fifteen years, but had increased more rapidly during the last three years. She had been treated almost exclusively by quacks, and during the last mentioned period had "suffered many things" from them.

I at once told her that there could be no hope from the effects of medicine, until the water was drawn off. She decided promptly that she would have the operation performed immediately. The patient was placed in a very large chair tilted far back, with a strong man to hold each end of the broad bandage around her.

The operation was performed in the usual manner; the water flowed freely, and she seemed to suffer very little until about eight gallons were drawn, when she began to grow faint and sick, and we were obliged to remove her to her bed and a recumbent posture. From this time, although she felt weak, she was not faint, but resolute and cheerful until the operation was completed.

Soon after getting on the bed she had a strong desire to urinate, and there was from that time an almost continued discharge of urine until the fluid ceased to flow from the canula.

After she got on the bed much of the fluid was lost, owing to her bad position, which from weakness and unwieldiness she was not able to change. Indeed the whole bed seemed saturated with it, yet what was saved and accurately weighed, amounted to one hundred and forty pounds.

After the evacuation of the fluid, the abdominal parietes, from long and excessive distention, on being drawn up by the bandage, formed large folds overlapping each other, and the sternum and short ribs stood out almost horizontally.

A bandage was kept applied as tight as could be borne with comfort, and within two weeks the parts assumed their natural position and appearance; the swelling of the lower extremities disappeared, and she has since enjoyed very tolerable good health without any very considerable appearance of dropsical enlargement.

